# Supplementary material for: The SAFFO Study: Sex-Related Prognostic Role and Cut-Off Definition of Monocyte-to-Lymphocyte Ratio (MLR) in Metastatic Colorectal Cancer
Source: Cancers (Basel). 2022 Dec 28;15(1):175. doi: 10.3390/cancers15010175 (PMC9818397; doi:10.3390/cancers15010175)
Supplement: Supplementary file 1 [file cancers-15-00175-s001.zip › cancers-2054953-supplementary.pdf]

Supplementary figure 1. Distributional graph of total lymphocytes and monocytes.

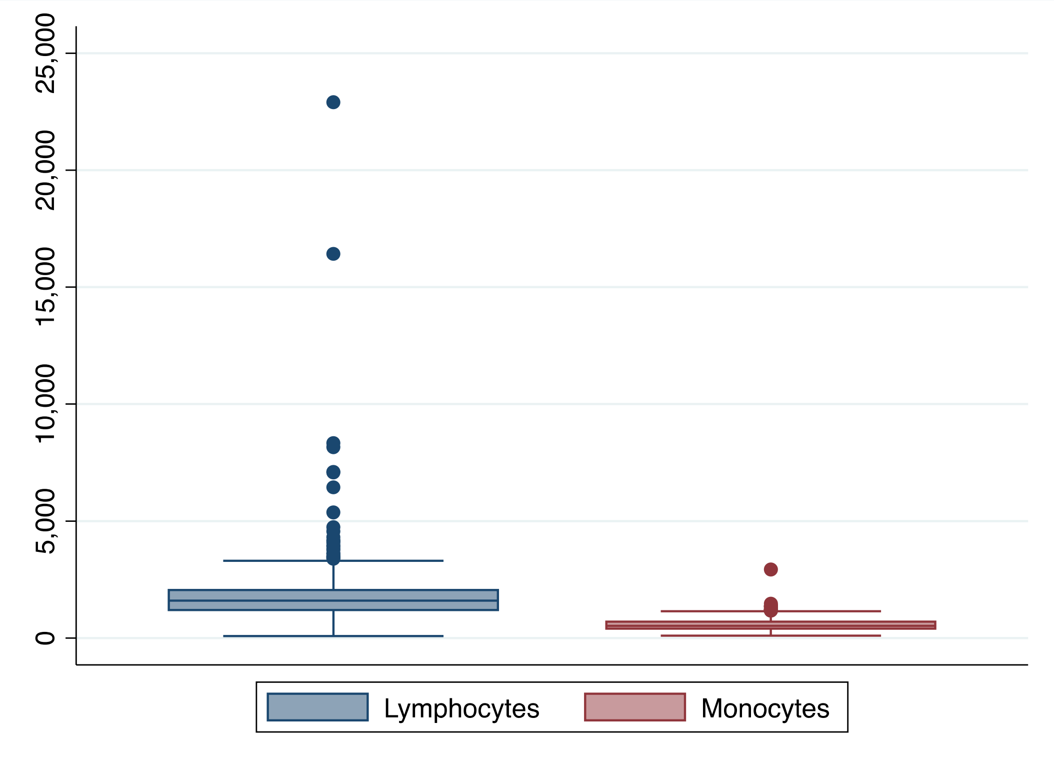

Supplementary table 1. Uni- and multivariate overall survival analyses in the training set

	Univariate analysis			Multivariate analysis		
**Variables**	**HR**	**P**	**95%CI**	HR	P	95%CI
MLR – Training set						
• **F > 0.27**	1.95	0.003	1.26-3.02	2.05	0.182	0.71-5.88
• **M ≤0.49**	1.31	0.206	0.85-2.02	1.15	0.774	0.42-3.18
• **M > 0.49**	2.65	0.010	1.26-5.59	4.70	0.020	1.27-17.42
KRAS						
• **Mut vs wt**	1.62	0.011	1.11-2.34	2.09	0.133	0.79-5.48
BRAF						
• **Mut vs wt**	2.24	0.028	1.08-4.60	6.53	0.002	1.98-21.57
**Sidedness**						
• **Right vs Left**	1.45	≤0.001	1.30-1.67	1.43	0.446	1.28-1.87
**Number of sites**						
• **>1 vs ≤1**	2.09	≤0.001	1.51-2.91	1.80	0.284	0.61-5.30
Sites of metastases						
• Lung **vs liver**	1.03	0.884	0.68-1.55	1.15	0.816	0.34-3.92
• Lymph nodes **vs liver**	1.58	0.114	0.89-2.79	0.52	0.428	0.10-2.57
• Peritoneum **vs liver**	3.05	≤0.001	1.98-4.69	5.13	0.012	1.44-18.28
• Bone **vs liver**	1.02	0.969	0.24-4.24	1.29	0.832	0.11-14.30
Surgery						
• Yes **vs No**	0.40	≤0.001	0.26- 0.63	0.11	0.032	0.11-0.90

Supplementary table 2. Uni- and multivariate overall survival analyses in the validation set

	Univariate analysis			Multivariate analysis		
**Variables**	**HR**	**P-value**	**95%CI**	HR	P-value	95%CI
MLR – Validation set						
• **F > 0.27**	2.21	0.010	1.21-4.06	3.31	0.012	1.29-8.46
• **M ≤0.49**	1.51	0.219	0.78-2.90	2.17	0.121	0.81-5.79
• **M > 0.49**	2.99	0.002	1.52-5.90	8.25	≤0.001	2.75-24.67
KRAS						
• **Mut vs wt**	1.07	0.664	0.77-1.48	1.58	0.106	0.90-2.75
BRAF						
• **Mut vs wt**	1.57	0.066	0.97-2.53	4.72	≤0.001	2.01-11.11
**Sidedness**						
• **Right vs Left**	1.56	≤0.001	1.40-1.88	1.25	0.405	0.80-1.78
**Number of sites**						
• **>1 vs ≤1**	1.73	≤0.001	1.27-2.37	0.46	0.034	0.22-0.94
Sites of metastases						
• Lung **vs liver**	1.03	0.885	0.63-1.70	0.57	0.175	0.25-1.28
• Lymph nodes **vs liver**	2.17	0.001	1.39-3.38	1.16	0.732	0.48-2.77
• Peritoneum **vs liver**	1.88	0.002	1.25-2.81	2.22	0.041	1.03-4.80
• Bone **vs liver**	2.06	0.123	0.82-5.18	0.79	0.684	0.25-2.42
• CNS **vs liver**	36.70	≤0.001	10.09-133.53	43.78	≤0.001	7.55-253.86
Surgery						
• Yes **vs No**	0.37	≤0.001	0.24-0.54	0.24	≤0.001	0.13-0.46
